# Deep Brain Stimulation for Addictive Disorders—Where Are We Now?

**DOI:** 10.1007/s13311-022-01229-4

**Published:** 2022-04-11

**Authors:** Jason Yuen, Abbas Z. Kouzani, Michael Berk, Susannah J. Tye, Aaron E. Rusheen, Charles D. Blaha, Kevin E. Bennet, Kendall H. Lee, Hojin Shin, Jee Hyun Kim, Yoonbae Oh

**Affiliations:** 1 Department of Neurologic Surgery, Mayo Clinic, Rochester, MN 55905 USA; 2 Deakin University, IMPACT, The Institute for Mental and Physical Health and Clinical Translation, School of Medicine, Geelong VIC 3216, Australia; 3 School of Engineering, Deakin University, Geelong VIC 3216, Australia; 4 Queensland Brain Institute, The University of Queensland, St Lucia, QLD 4072 Australia; 5 Department of Psychiatry & Psychology, Mayo Clinic, Rochester, MN 55905 USA; 6 Department of Psychiatry, University of Minnesota, Minneapolis, MN 55455 USA; 7 Department of Psychiatry, Emory University, Atlanta, GA 30322 USA; 8 Division of Engineering, Mayo Clinic, Rochester, MN 55905 USA; 9 Department of Biomedical Engineering, Mayo Clinic, Rochester, MN 55905 USA

**Keywords:** Deep brain stimulation, Addiction, Biomarkers, Animal models, Neuromodulation, Neuropsychiatry

## Abstract

In the face of a global epidemic of drug addiction, neglecting to develop new effective therapies will perpetuate the staggering human and economic costs of substance use. This review aims to summarize and evaluate the preclinical and clinical studies of deep brain stimulation (DBS) as a novel therapy for refractory addiction, in hopes to engage and inform future research in this promising novel treatment avenue. An electronic database search (MEDLINE, EMBASE, Cochrane library) was performed using keywords and predefined inclusion criteria between 1974 and 6/18/2021 (registered on Open Science Registry). Selected articles were reviewed in full text and key details were summarized and analyzed to understand DBS’ therapeutic potential and possible mechanisms of action. The search yielded 25 animal and 22 human studies. Animal studies showed that DBS of targets such as nucleus accumbens (NAc), insula, and subthalamic nucleus reduces drug use and seeking. All human studies were case series/reports (level 4/5 evidence), mostly targeting the NAc with generally positive outcomes. From the limited evidence in the literature, DBS, particularly of the NAc, appears to be a reasonable last resort option for refractory addictive disorders. We propose that future research in objective electrophysiological (e.g., local field potentials) and neurochemical (e.g., extracellular dopamine levels) biomarkers would assist monitoring the progress of treatment and developing a closed-loop DBS system. Preclinical literature also highlighted the prefrontal cortex as a promising DBS target, which should be explored in human research.

**Supplementary Information:**

The online version contains supplementary material available at 10.1007/s13311-022-01229-4.

## Introduction

**Fig. 1:**
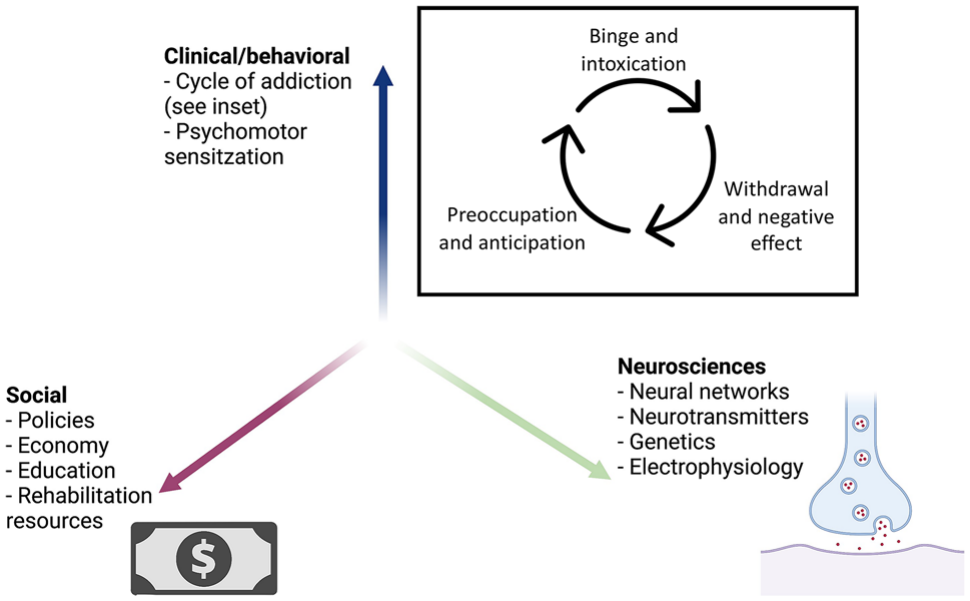
Biopsychosocial model depicting the different dimensions in consideration of addiction treatment. The stages of the addiction cycle of behavior are shown (inset) [140]. Interactions between components in different domains lead to addictive behaviors. Created with Biorender.com

Addictive substances pose significant socioeconomic impact on society and the healthcare system, costing hundreds of billion dollars annually in the USA alone [1, 2]. Psychological and adjunctive pharmacological treatments are the current mainstay for addiction therapeutics. However, the risk of relapse remains high, with some citing relapse rates as high as 75 to 98% within 1 year of treatment [3]. Investigations into new treatment modalities are urgently warranted. To develop novel therapies for addiction, researchers are focusing on the behavioral and neuroscience aspects in the biopsychosocial model (Fig. 1). Deep brain stimulation (DBS) in particular is a neuroscience-based potential treatment for medically refractory addictive disorders, given its diminishing surgical risks. “Medically refractory” cases have no formal definition, although these are generally regarded when conventional treatments repeatedly fail [4].

Recent advances in neuromodulation techniques and devices led the indication of DBS to include movement disorders, such as Parkinson’s disease, to neuropsychiatric conditions, such as depression, Tourette’s syndrome, and obsessive–compulsive disorder (OCD) [5–8]. The use of DBS to reduce substance use has also been explored in both preclinical and clinical settings [9–12], offering a beacon of hope to patients suffering from medically refractory addictive disorders. This review aims to summarize and evaluate the potential therapeutic efficacy, mechanisms of action, and biomarkers involved in the use of DBS in the treatment of addictive disorders. We cautiously report that DBS is promising to treat certain refractory addictive disorders. Recommendations for the future directions are also provided.

## Addiction Neurobiology: a Brief Overview

**Fig. 2:**
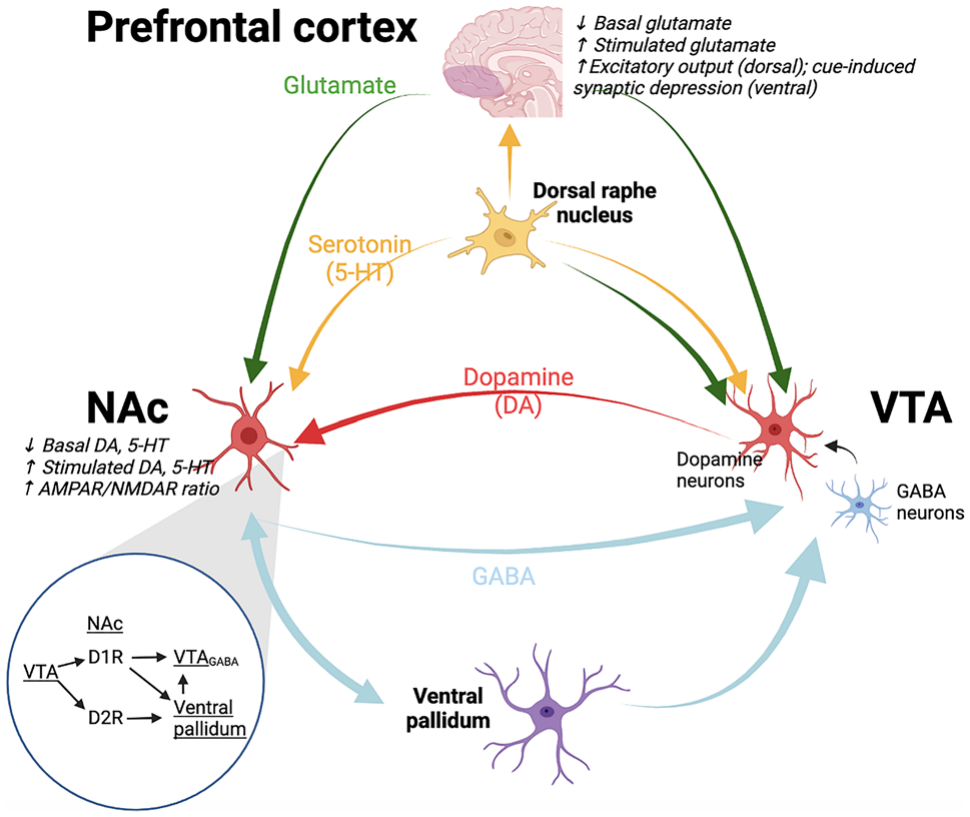
A simplified illustration of the neurotransmitters and neuroanatomical structures involved in the pathophysiology of addiction [13, 19, 20]. Other relevant areas are omitted for clarity. The effect of dopamine receptors D_1_-R and D_2_-R is shown in inset. D_1_-R mediates the direct pathway (positive reinforcement) and D_2_-R mediates the indirect pathway (negative reinforcement). *Italic* descriptions denote changes secondary to chronic drug use. 5-HT, serotonin; AMPAR, α-amino-3-hydroxy-5-methyl-4-isoxazolepropionic acid receptor; D1/2R, dopamine 1/2 receptors; DA, dopamine; GABA, gamma aminobutyric acid; NAc, nucleus accumbens; NMDAR, N-methyl-d-aspartate receptor; VTA, ventral tegmental area. Created with Biorender.com

Addiction is a highly complex process involving multiple neurochemicals and brain structures. A highly simplified model highlights the central role of dopamine in mediating drug reward learning and drug seeking (compulsive) behaviors (Fig. 2). In essence, addictive drugs, such as the psychostimulants and opioids, enhance synaptic concentrations of dopamine in forebrain subcortical structures, such as the nucleus accumbens (NAc) [13]. These drugs of abuse significantly enhance dopamine concentrations over and above the ability of natural rewards, hijacking the system and encoding a more biologically salient reward association with surrounding environmental cues [13]. Such Pavlovian drug-environmental conditioning can trigger the desire to seek more of the drug. For example, the drug associated cues can elicit a surge of dopamine, which may manifest in drug craving, seeking and ultimately use [14].

Serotonin (5-HT) is another important neurotransmitter mediating neuroplasticity, hedonic tone, motivational, and reinforcement processes, as well as cognitive functions [15, 16]. 5-HT_2A_ and 5-HT_2C_ receptors in the prefrontal cortex (PFC), NAc, and ventral tegmental area in particular appear to be critical. 5-HT_2A_ receptor increases, and 5-HT_2C_ receptor decreases, striatal dopamine release [15]. The serotonergic neuroadaptations may be accountable for emotional components such as anhedonia and depression during drug withdrawal [16]. Recent evidence in mice also suggest that serotonin signaling via 5-HT_1B_ receptor may attenuate transition from casual to compulsive cocaine use [17].

Chronic drug use ultimately alters the homeostatic neuroplasticity in the brain which not only results in a sensitized dopaminergic system [18], but also leads to changes in the glutamatergic system [13, 19, 20]. For example, calcium-permeable ionotropic glutamatergic AMPA receptors become upregulated relative to glutamatergic NMDA receptors [13, 19, 20]. The changes in AMPA and NMDA receptors have become a hallmark process of neuroplasticity that underpins behavioral changes following chronic drug use [21]. Another neurobiological adaptation that occurs with chronic drug use is the process of “dorsalization” [22]. Here, repeated exposure to drugs of abuse leads to recruitment of the circuit that projects from the orbitofrontal cortex (OFC) to the dorsal striatum (caudate and putamen), where synaptic potentiation occurs at both dopamine receptors 1 and 2 (D_1_-R and D_2_-R) on GABAergic medium spiny neurons (MSNs).

Activation of D_1_-Rs receptors causes excitation in the postsynaptic cell via cyclic adenosine monophosphate production (cAMP) production [23] that ultimately leads to promotion of long-term potentiation [24]. D_1_-R- and NMDA receptor–dependent activation of extracellular signal-regulated kinase (ERK) in brain areas such as striatum is an important pathway for drug-induced locomotor response and sensitization [25–28]. For example, systemic injection of D_1_-R- or NMDA antagonist prevents ERK phosphorylation triggered by d-amphetamine in the mouse striatum [29]. In addition, cocaine-induced ERK phosphorylation and locomotor sensitization is prevented with mitogen-activated protein kinases/ERK kinase inhibitor or in mutant mice with alanine-replaced Thr-34 residue of dopamine- and cAMP-regulated phosphoprotein (DARPP-32) [29]. D_1_-R MSN structural plasticity depends on NMDA and DARPP-32 signaling [24]. Psychostimulants appear to affect ERK signaling via DARPP-32-based D_1_-R- and NMDA intracellular pathways [29].

Importantly, D_1_-R activation inhibits long-term depression, as revealed by D_1_-R antagonist unmasking long-term depression [30]. In contrast, D_2_-Rs can promote long-term depression and prevent the induction of long-term potentiation [30]. However, D_2_-Rs are particularly sensitive to dips in the tonic dopamine level, during which they activate protein kinase A (PKA) to induce long-term potentiation [30–32]. Taken together, D_1_-R and D_2_-R are critical for bidirectional plasticity of striatal MSNs when dopamine levels are high vs low to guide adaptive and maladaptive behaviors involved in reward learning [31, 32]. Therefore, D_1_-R and D_2_-R signaling dynamically modulates cortico-striatal plasticity depending on the level of synaptic dopamine to facilitate transition into persistent drug seeking and use.

While DBS appears to target these processes involved in addiction to reduce drug seeking and taking [10, 33–37], the potential therapeutic efficacy and mechanisms of action of DBS in the treatment of addictive disorders are poorly understood. We address this gap in the present review.

## Methods

**Fig. 3:**
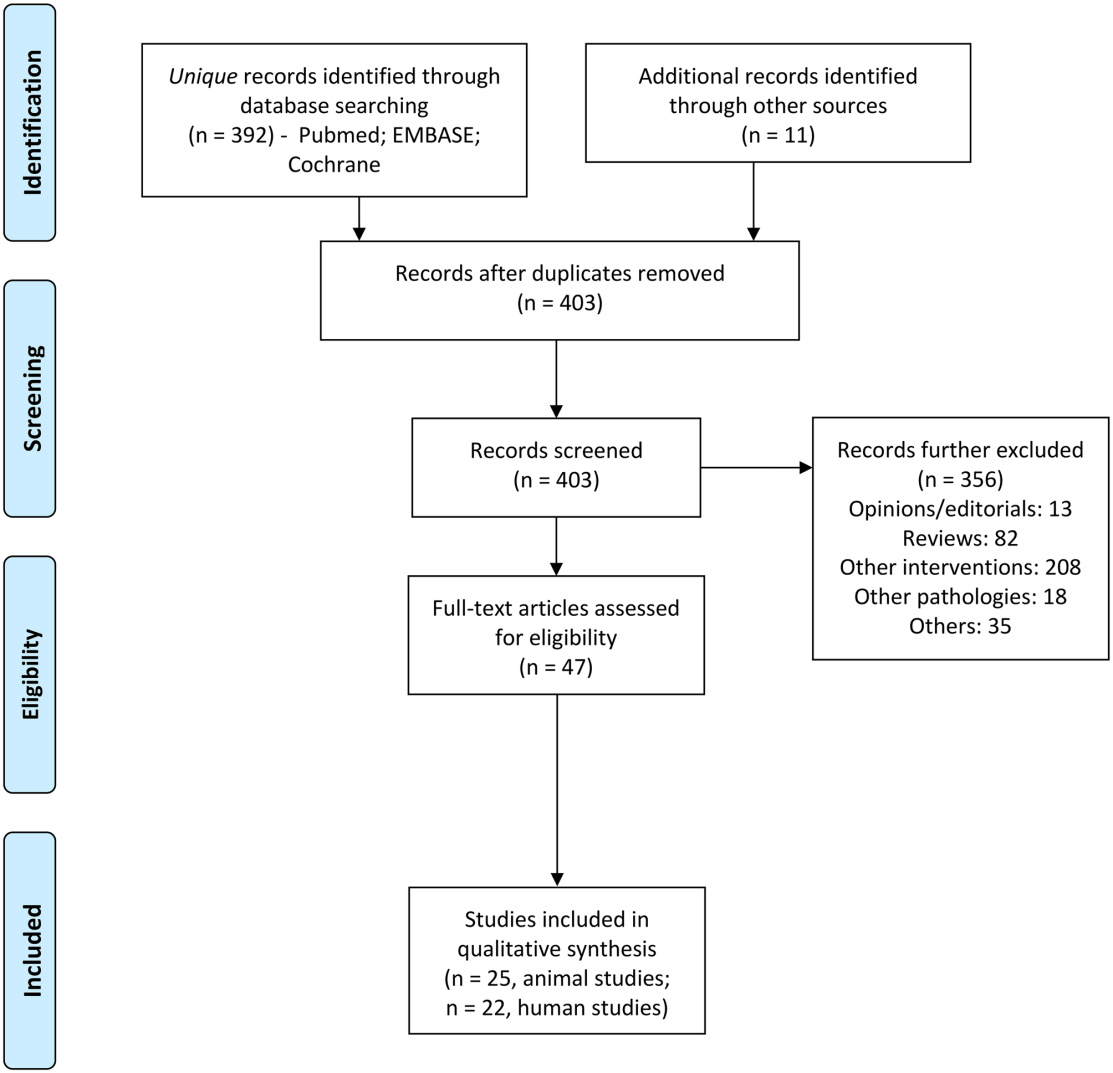
Preferred Reporting Items for Systematic Reviews and Meta-Analyses (PRISMA) flow chart of literature search. Other sources include those found by reviewing the references from included papers

A search was performed using keywords, such as “deep brain stimulation” and “addiction” in MEDLINE, EMBASE, and Cochrane Library database between 1974 and 6/18/2021. Details of search criteria and inclusion and exclusion criteria are given in Supplementary Information Appendix A. Search results are presented in a Preferred Reporting Items for Systematic Reviews and Meta-Analyses (PRISMA) flow chart (Fig. 3). This review was registered at the Open Science Framework [38]. The process yielded 47 papers (25 preclinical studies and 22 human studies), which were further reviewed in full text. The results of this literature search are summarized in the sections below.

## Results and Discussion

### Preclinical Models and Clinical Correlation

**Fig. 4:**
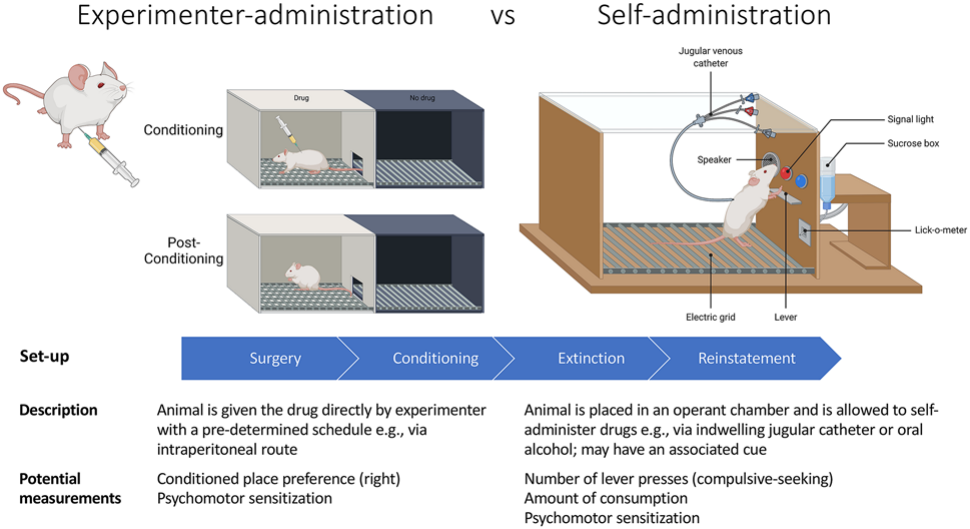
Illustration of two most widely used experimental paradigms for the study of drug addiction in animals. On the left, conditioned place preference (CPP) is shown, where “non-contingent” drug-administration is associated with one side of the chamber during drug-side associated conditioning. If the drug is experienced as rewarding, the animal will spend more time in the drug-administered side relative to the other side, even in the absence of further drug administration. On the right, “contingent” drug self-administration paradigm allows the animal to regulate its own drug intake (e.g., via nose-poke or lever-pressing). A discrete light and/or tone cue may be provided in association with the drug intake to serve as a drug-associated cue. In extinction, the animal is placed back into the environment where CPP or self-administration is acquired but without drug availability. Extinction sessions lead to reduced place preference or drug seeking. During reinstatement, the drug, the drug-associated cue, and/or stress can be given, which can lead to “relapse” measured by preference or drug seeking. Created with Biorender.com

Animal DBS studies in the selected literature (Table 1) predominantly used conditioned place preference (CPP) or self-administration paradigms (Fig. 4) to model addiction. Self-administration models have high construct, content, and face validity relative to human addictive behaviors. Experimenter-administration models such as CPP can measure drug-induced behavioral sensitization, such as increased locomotion, and are easier to perform. Both models also allow modeling of withdrawal symptoms during abstinence and/or extinction phase, followed by relapse behaviors, such as cue or drug-induced reinstatement. Most common DBS brain targets in the selected animal studies (Table 1) were the NAc [33, 39–45], PFC [44, 46, 47], and subthalamic nucleus (STN) [10, 37]. The following sections will focus on these three most studied targets.

**Table 1:** Summary of animal studies in deep brain stimulation and addiction, listed in chronological order. *ACC*, anterior cingulate cortex; *BLA*, basolateral amygdala; *CPP*, conditioned place preference; *EA*, experimenter-administration; *EtOH*, alcohol (ethanol); *FR*, fixed ratio; *GABA*, γ–aminobutyric acid; *HFS*, high-frequency stimulation; *i.p.*, intraperitoneal injection; *LFS*, low-frequency stimulation; *LH*, lateral hypothalamus; *(m)PFC*, (medial) prefrontal cortex; *NAc*, nucleus accumbens; *OD*, once daily; *OFT*, open field test; *PAG*, peri-aqueductal gray; *pCREB*, phosphorylated cyclic AMP-response element binding protein; *SA*, self-administration; *s.c.*, subcutaneous injection; *STN*, subthalamic nucleus; *vHipp*, ventral hippocampus; *VTA*, ventral tegmental area; *NA*, data not available

Author and year	Species and strain (sample size)	Target; laterality	Behavioral paradigm	Stimulation parameters				Main outcomes
				Hz	Pulse width	Current	DBS session	
Pinel et al. (1975) [85]	Black-hooded male rats (*N* = 27)	Amygdaloid complex; unilateral (side unspecified)	Alcohol (1000–2018 mg/kg ethanol) via experimenter intubation	60	NA	400 μA	45 stimulations (1 s duration), 3 × /day, 5 days/week	Stimulation was performed to induce kindling (until displaying at least 1 seizure), and alcohol was administered after stimulation. The withdrawal syndrome observed 9 h following alcohol administration in kindled (stimulated) animals was much more *severe* than that of the controls
Beaulieu and Thorn (1986) [54]	Long-Evans male rats (*N* = 29)	PAG; midline target	Subcutaneous morphine 72 mg pellet (for 72 h) vs placebo	50	NA	20–200 μA	30 min after naloxone-precipitated withdrawal	Large range of current applied to different rats (depending on analgesia threshold) Stim. attenuated morphine withdrawal behaviors, esp. the recessive behaviors associated with autonomic changes (e.g., eye-twitching) lasting 2 h or more
Levy et al. (2007) [35]	Sprague–Dawley male rats (*N* = 7–14/group)	Lateral hypothalamus (100 Hz) or PFC (20 Hz, 100 Hz); bilateral	Cocaine (1 mg/kg reducing to 0.5 mg/kg) self-administration. Progressive ratio was also assessed at 0.5 mg/kg Locomotion following cocaine challenge 15 mg/kg (i.p.)	20, 100	100 μs	200–400 μA	30 min/day for 10 days	DBS (100 Hz) in lateral hypothalamus or PFC reduced cocaine-, but not sucrose seeking. DBS (100 Hz) in the PFC also reduced cocaine taking in a progressive ratio self-administration test Cocaine-induced locomotor hyperactivity was exaggerated by lateral hypothalamus DBS but was reduced by PFC DSB The behavioral findings were accompanied by GluR1 alterations in the NAc (increased) and the VTA (variable depending on subregion)
Liu et al. (2008) [39]	Sprague–Dawley rats of unspecified sex (*N* = 32)	NAc core; right	CPP induced by i.p. morphine 10–60 mg/kg (increasing dose)	130	210 μs	200–500 μA	3 h (15-min trains with 45-min intervals)	Pre-conditioning DBS sessions prevented morphine-induced CPP compared to sham
Vassoler et al. (2008) [40]	Sprague–Dawley male rats (*N* = 7–12/group)	NAc shell or dorsal striatum; bilateral	Cocaine self-administration FR1 (250 μg/kg per infusion) progressing to FR5	160	60 μs	150 μA	Continuous during 2-h reinstatement sessions	DBS in NAc immediately following 10 or 20 mg/kg cocaine prime attenuated cocaine-induced reinstatement compared to sham DBS. DBS in dorsal striatum had no effects on reinstatement
Knapp et al. (2009) [51]	Long-Evans male rats (*N* = 4–5/group)	NAc shell or NAc core (2 groups); bilateral	Alcohol (0–10% ethanol) self-administration	160	200 μs	0–150 μA	5 min	At 150 μA, but not at 100 μA or less, DBS in shell or core given prior to established alcohol self-administration sessions reduced alcohol consumption
Friedman et al. (2010) [58]	Sprague–Dawley male rats (*N* = NA)	Lateral habenula; right	Cocaine self-administration 0.25, 0.5, and 1 mg/kg i.v. for 21 days Reinstatement: 0, 5, 10, 20 mg/kg i.p	10–100	500 μs	200 μA	For 15 min at start of task	LFS alone appeared to have superior effect on seeking behavior compared to HFS With DBS consisting of a combination between 10 and 100 Hz, drug seeking behavior was reduced in self-administration, extinction and relapse phases Lesioning actually increases drug seeking behavior (increased number of presses during extinction) Tests controlled by forced swim test (depression-like behavior), open-field test (locomotion), etc Western blot showed elevated glutamatergic receptor subunits NR1 and GluR1 and scaffolding Protein PSD95, but not GABA Ab, protein levels in VTA with cocaine SA but normalized after DBS DBS reduced cocaine seeking behavior during both self-administration and extinction training, as well as lever-pressing behavior during reinstatement
Henderson et al. (2010) [52]	Alcohol-preferring male rats (*N* = 20)	NAc shell; bilateral	Home cage alcohol (10% ethanol) self-administration	140–150	60 μs	200 μA	1 h on 1 h off DBS stage 2: 24 h when free access was allowed again	DBS given prior to established alcohol self-administration sessions reduced alcohol consumption and preference over water. DBS given prior to alcohol-induced relapse following 4–6 weeks of abstinence also reduced alcohol consumption and preference over water
Rouaud et al. (2010) [59]	Long-Evans male rats (*N* = 88)	STN; bilateral	Cocaine self-administration FR 1 (0.25 mg per infusion) Progressive ratio was also assessed at 0.25 mg per infusion CPP induced by cocaine (10 mg/kg i.p.)	130	60 μs	50–130 μA	DBS was applied throughout each behavioral session	DBS decreased cocaine but not food taking Progressive ratio tests showed that DBS decreases motivation to attain cocaine but increased motivation to attain food. Similar dissociation was observed with CPP, with DBS reducing cocaine CPP but increasing food CPP
Guo et al. (2013) [41]	Sprague–Dawley male rats (*N* = 54)	NAc core; mixed unilateral or bilateral	Heroin self-administration FR1 (50 μg/kg per infusion) paired with a light cue	130	100 μs	0, 75, or 150 μA	1-h continuous stimulation	Daily DBS (75 and 150 μA) sessions during abstinence reduced subsequent cue- and heroin-induced drug seeking, except for DBS only in the left NAc. DBS increased pCREB but decreased ΔFosB in NAc core and shell DBS did not affect locomotion, nor spatial learning and memory
Ma et al. (2013) [55]	Sprague–Dawley male rats (*N* = 25)	NAc shell; bilateral	Morphine EA 5 mg/kg i.p. progressing to 90 mg/kg i.p. over 12 days Reinstatement—2.5 mg/kg i.p	130	60 μs	2 V (stim. voltages)	60 min, 5 × /day for 30 days	CPP training was first given with increasing doses of morphine; naloxone then given to precipitate “withdrawal.” DBS prevented drug seeking behavior (in terms of CPP measurements) during re-instatement of morphine Place navigation studies were performed (Morris water maze) to assess learning and memory—no change by DBS
Pushparaj et al. (2013) [53]	Sprague–Dawley male rats (*N* = 6–12/group)	Insular cortex; bilateral	Nicotine SA 0.03 mg/kg i.v. per infusion under a FR-5 then progressive ratio schedule Reinstatement dose was 0.15 mg/kg s.c	130	90 μs	50 μA	HFS started from 5 min before behavioral sessions and lasted throughout the sessions	DBS significantly attenuated nicotine taking, under both FR5 and progressive ratio schedules, as well as nicotine seeking behavior induced by cues and priming; no effect on food taking behavior HFS of brain slices containing the insular region was found to inactivate insular neurons
Vassoler et al. (2013) [42]	Sprague–Dawley male rats (*N* = 5–12/group)	NAc shell or core; bilateral	Cocaine self-administration FR1 (250 μg/kg per infusion) progressing to FR5	160	60 μs	150 μA	Continuous during 2-h reinstatement sessions	DBS of NAc shell, but not core, immediately following 10 mg/kg cocaine prime attenuated cocaine-induced reinstatement compared to sham DBS (0 μA) DBS of NAc shell induced *c-Fos* expression locally and in the infralimbic cortex
Wilden et al. (2014) [34]	Alcohol-preferring female rats (*N* = 7)	NAc shell; unilateral	Alcohol (15% ethanol) self-administration	150	100 μs	100 or 200 μA	65 min	DBS (200 μA but not 100 μA) starting 5 min prior to and throughout established daily alcohol self-administration sessions reduced alcohol taking. Alcohol taking resumed at baseline levels post DBS sessions
Creed et al. (2015) [33]	C57BL/6 transgenic male mice (*N* = 6–12/group)	NAc shell; bilateral	Locomotor sensitization induced by cocaine 20 mg/kg i.p	12 or 130	90 μs	50 μA	60 min	DBS at 130 Hz, but not at 12 Hz, given immediately before test transiently suppresses locomotor sensitization. DBS had no behavioral effects when applied 4 or 24 h before test DBS at 130 Hz 24 h before did not affect cocaine-evoked plasticity DBS at 12 Hz in combination with a D_1_-R antagonist reversed cocaine-induced hyperlocomotion and plasticity. DBS at 12 Hz had no effects by itself
Hamilton et al. (2015) [43]	Long-Evans male rats (*N* = 6–8/group)	NAc; right hemisphere	Cocaine self-administration FR1 (500 μg/kg per infusion) progressing to FR3	20 or 160	100 μs	50–200 μA	30 min	A single DBS session did not affect cocaine taking. Daily DBS 20 or 160 Hz sessions during 14 days of abstinence led to attenuated cocaine seeking following a 5 mg/kg cocaine prime when tested 1 day, but not 14 days, after the last DBS session
Mehdipour et al. (2015) [46]	Wistar male rats (*N* = 6 in each group)	Prelimbic cortex; right hemisphere	Light–dark shock passive avoidance reversed by morphine (10, 20, or 40 mg/kg) i.p	60	NA	25, 50, 100, or 150 μA	20 min (duration of 1 s every 5 s)	Methodology was unclear (e.g., interval between doses and shock). Short-term stimulation was given DBS at 50 and 100 μA stimulation reversed passive avoidance memory attenuation by morphine. DBS at 25 and 150 μA had no effects
Hadar et al. (2016) [44]	Wistar male rats (*N* = 6–15/group)	NAc shell, infralimbic cortex, or striatum (caudate, putamen); bilateral	Home cage alcohol (5, 10, or 20% ethanol) and water two bottle choice self-administration	130	90 μs	5.25 V (stim. voltages)	24 h or 4 days	Compared to sham DBS, 4 days of NAc shell DBS led to augmented alcohol-induced relapse following chronic abstinence. Acute (24 h) DBS reduced alcohol-induced relapse. DBS in infralimbic cortex or striatum had no effects on behavior. Acute NAc DBS (24 h) increased local dopamine levels in alcohol-exposed rats
Martínez-Rivera et al. (2016) [56]	Sprague–Dawley male rats (*N* = 87)	Ventral capsule/ventral striatum (VS); bilateral	Morphine EA 5 mg/kg s.c./day for 4 days with CPP training	20 or 130	100 μs	100–200 μA	60 min/day over 9 days of extinction	HFS of dorsal VS impaired extinction training and extinction memory but no effect for HFS of ventral VS LFS of dorsal VS strengthened extinction memory when tested 2 or 9 days after the cessation of stimulation Both HFS and LFS increased *c-Fos* expression in infralimbic PFC, but only LFS increased *c-Fos* in the basal amygdala and the medial portion of the central amygdala
Batra et al. (2017) [45]	Wistar male rats (*N* = 18)	NAc shell; bilateral	Methamphetamine self-administration FR1 (0.05 mg/kg per infusion)	130	60 μs	200 μA	3 h	The first two daily sessions of DBS prior to self-administration sessions reduced methamphetamine taking compared to sham DBS, while the remaining 3 DBS sessions had no effects. These DBS sessions reduced cue-induced reinstatement when tested 20–28 days later
Wade et al. (2017) [37]	Wistar male rats (*N* = 32)	STN; bilateral	Heroin SA 60 μg/kg i.v FR1 3- or 12-h sessions 5 days per week	130	60 μs	50–130 μA	Throughout the SA sessions	STN HFS attenuated re-escalation of heroin intake post-abstinence in rats with extended access to heroin STN HFS inhibited substantia nigra, entopeduncular nucleus, and NAc shell (measured with brain mapping analyses of immediate-early gene expression) but not PFC, and silenced STN neurons (measured using recording ex vivo)
Chang et al. (2020) [57]	Sprague–Dawley male rats (*N* = 9–15/group)	Anterior insular cortex; bilateral	CPP induced by subcutaneous injection of morphine (10 mg/kg)	130	90 μs	150 μA	11 or 14 days during extinction or abstinence	14 days of DBS during abstinence attenuated the expression of morphine-CPP but CPP relapsed 10 days after the cessation of DBS. Eleven days of DBS during CPP extinction facilitated extinction acquisition as well as alleviating morphine-induced reinstatement OFT and novel object recognition test showed no impairment to locomotion and recognition memory Proteomic analysis revealed the expression levels of 8 morphine-regulated proteins in the anterior insula were reversely changed by HF-DBS
Fakhrieh-Asl et al. (2020) [9]	Wistar male rats (*N* = 108)	Orbitofrontal cortex; bilateral	CPP induced by subcutaneous injections of morphine 3–7 mg/kg (increasing dose)	13 or 130	100 μs	150 μA	20 or 40 min, once daily	HFS but not LFS given daily for 3 days of conditioning significantly prevented CPP acquisition HFS but not LFS given daily for 6–10 days of extinction facilitated extinction acquisition and blocked morphine-induced reinstatement DBS did not cause any changes in locomotor activity, novel objection recognition memory, nor anxiety-like behavior
Guercio et al. (2020) [47]	Sprague–Dawley male rats (*N* = 6–8/group)	Infralimbic cortex, prelimbic cortex, anterior cingulate cortex, BLA or vHipp; bilateral	Cocaine self-administration FR1 (250 μg/kg per infusion) progressing to FR5	160	60 μs	150 μA	Continuous during 2 h reinstatement sessions	DBS at BLA, vHipp, and infralimbic cortex immediately following 10 mg/kg cocaine prime attenuated cocaine-induced reinstatement compared to sham DBS (0 μA), but BLA and vHipp also attenuated sucrose reinstatement Anterior cingulate cortex and prelimbic cortex DBS did not attenuate either
Degoulet et al. (2021) [10]	Male Lister Hooded rats (*N* = 84)	STN; bilateral	Cocaine SA FR1 progressing to random interval until 120 s Punishment sessions (foot shock) also applied (rats divided into shock-resistant and shock-sensitive subgroups)	8, 30, or 130	80 μs	50–150 μA	DBS turned on and intensity was increased to reach predetermined parameters prior to start of behavioral session, and continued during session	30 Hz but not 130 Hz DBS reduced pathological cocaine seeking during treatment, whereas 130 Hz actually increased seeking behavior acutely Compulsive, i.e., shock-resistant rats exhibit pathological STN low-frequency oscillations (LFP) during cocaine escalation (especially < 20 Hz)

### NAc Electrical Stimulation

The NAc is divided into core and shell subregions with different histopathological compositions, projections, and functions [48]. Studies suggest that the *shell* is responsible for reinforcing properties of novelty, rewards (both drug- and non-drug-related), and drug relapse, whereas the *core* mediates spatial learning, conditioned responses, responses to motivational stimuli, and impulsive choices [14, 49]. These features of motivated behaviors suggest NAc shell underlying extinction and reinstatement behavior [50], and NAc core underlying the initial acquisition of drug taking and cue-elicited drug seeking [48]. However, there is synergy between the two subnuclei, and both play a role in drug addiction.

Stimulation of either the core or shell subregions of the NAc using a range of low and high frequencies leads to reduced drug taking, seeking, or CPP in rats conditioned with alcohol [34, 51, 52], nicotine [53], opioids [9, 37, 39, 41, 46, 54–57], cocaine [10, 33, 35, 40, 42, 43, 47, 58, 59], or methamphetamine [45] (Table 1). Behavioral improvement was related to the timing of when electrical stimulation was given—that is, stimulation given before a self-administration session or CPP acquisition would reduce drug intake or prevent CPP, respectively [39, 45]. When given during abstinence or reinstatement, stimulation would diminish drug or cue-induced drug seeking after extinction and/or forced abstinence phase [40–43, 45]. Such efficacy of stimulation during abstinence to prevent relapse is particularly promising in the clinical context.

While most animal and human studies (Table 2) show success in modulating addictive behavior using NAc stimulation, there are *two main issues*. First, there is conflicting evidence in animal studies regarding the efficacy of NAc core vs shell stimulation. Both high- and low-frequency stimulation (HFS, LFS) of NAc (electrodes placed across core and shell) can reduce cocaine-primed drug seeking following 24 days of abstinence [43]. Other studies using core as the DBS target showed reduced morphine-induced CPP [39], ethanol self-administration [51], and heroin reinstatement [41]. The shell was also shown to be an effective target for morphine-induced CPP [55], cocaine reinstatement [40], ethanol self-administration [44, 51, 52], and methamphetamine reinstatement [45]. In contrast, Vassoler et al. demonstrated that stimulation of the NAc shell but not the core attenuated cocaine-induced reinstatement [42].

**Table 2:** Summary of human studies, listed in chronological order. *ACC*, anterior cingulate cortex; *ALIC*, anterior limb of internal capsule; *AMP*, amphetamine; *BNST*, bed nucleus of stria terminalis; *BZ*, benzodiazepine; *CT*, computed tomography; *EEG*, electroencephalogram; *EtOH*, alcohol; *F*, female; *LFP*, local field potential; *M*, male; *MRI*, magnetic resonance imaging; *NAc*, nucleus accumbens; *OCD*, obsessive–compulsive disorder; *PD*, Parkinson’s disease; *PET*, positron-emission tomography; *QoL*, quality of life; *STN*, subthalamic nucleus; *VC/VS*, ventral capsule/ventral striatum; *NA*, data not available

Author and year	Indication for DBS (*N; age sex*)	Target	Drug	Stimulation parameters			Laterality	Adverse events	Outcome
				Hz	μs	V			
Witjas et al. (2005) [82]	PD (2; 53 M, 38 M)	STN	Dopaminergic agent (despite severe dyskinesia)	*High*	NA	NA	Bilateral	None reported	Drugs stopped or much reduced immediately post-operation At 2 years’ follow-up, continue to be show benefit Dyskinesia and mood also much improved with no change in cognition
Kuhn et al. (2007) [141]	Addiction (1; 54 M)	NAc	EtOH	130	90	4.5 V	Bilateral	None reported	Comorbid anxiety and depressive disorders Reported feeling of “inner appeasement” acutely After 12 months of treatment, the alcohol consumption and craving reduced dramatically
Kuhn et al. (2009) [93]	Tourette’s syndrome (4), OCD (5) or anxiety disorders (1) (10; 7 M and 3F, range from 28–58 years)	NAc	Nicotine	130–145	90, 180	3–6 V	5 bilateral, 5 unilateral	None reported	Fagerström Test for Nicotine Dependence was used to assess smoking behavior At 30-month follow-up, 3 out of 10 stopped smoking but the others mostly had no changes Those who quit smoking had higher stimulating voltages but difficult to compare due to small numbers
Mantione et al. (2010) [94]	OCD (1; 47F)	NAc	Nicotine	185	90	3.5 V	Bilateral	None reported	Comorbid mild anxiety and depression Dramatic improvement in OCD, anxiety, and depression Smoking cessation and weight loss was observed after 10 months post-operation (up to at least 2-year follow-up)
Kuhn et al. (2011) [78]	Addiction (1; 69 M)	NAc	EtOH	130	120	5.5 V	Bilateral	None reported	Abstinence after 1 year of treatment Improvement seen in psychometric score EEG showed enlargement in error-related negativity (amplitude when error occurs during test) and reduction in error rate
Zhou et al. (2011) [90]	Addiction (1; 24 M)	NAc	Heroin	145	90	2.5 V	Bilateral	Transient confusion and urinary incontinence initially	Abstinent for 6 years from implantation (removed after first 3 years) Improvement in cognition and memory
Heldmann et al. (2012) [92]	Addiction (1; 38 M)	NAc	EtOH	130	90	3.5 V	Bilateral	None reported	Risk taking behavior was assessed by gambling task—less risk taking behavior when DBS was switched on, compared to off-state PET showed activations in the paracingulate cortex, temporal poles, precuneus and hippocampus with active DBS Except for the temporal pole, these activations were not seen when DBS was off
Valencia-Alfonso et al. (2012) [142]	Addiction (1; 47 M)	NAc	Heroin	180	90	3.5 V	Bilateral	None reported	Mostly clean for 6 months apart from self-limiting relapse (although activation of some contacts initially increased craving) Intracranial EEG showed higher power for drug-related compared with non-drug-related cues in the gamma-band (40–60 Hz), which also has a lower correlation to frontal theta signal compared with the drug-unrelated responses on the right side
Voges et al. (2013) [143] Cases overlap with Muller et al. (2009) [144] and Heinze et al. (2009) [145]	Addiction (5; all male, range from 36–65 years)	NAc	EtOH	130	90	3.5 V	Bilateral	Transient hypomania	Average follow-up was 38 months LFP using externalized electrodes showed error-related modulations at NAc during neuropsychological task and error-related negativity obtained from NAc preceded the surface error-related activity All subjects experienced significant improvement of craving. Two remained completely abstinent for more than 4 years
Kuhn et al. (2014) [67]	Addiction (2; 33 M, 31F)	NAc	EtOH and AMP, AMP and BZ	140 130	120 90	5 V 4.5 V	Bilateral	1 seizure (known epilepsy)	Improved depressive and anxious symptoms, as well as consumption and craving in methadone and heroin Patients started to consume other psychotropic substance and continued to consume amphetamine out of “boredom” and for weight-related reason without subjective craving
Gonçalves-Ferreira et al. (2016) [146]	Addiction (1; 36 M)	NAc, BNST, ALIC	Cocaine	130	150	2.5–4 V	Bilateral	Only transient, e.g., metallic taste, warm sensation at high voltages	Previous comorbid heroin addiction DBS at ALIC showed no efficacy but posterior NAc and BNST appeared to be effective targets After 2.5 years, including a blinded on/off period, DBS resulted in improvement in cocaine dependence both objectively and subjectively
Müller et al. (2016) [11] Cases (3) overlap with Muller et al. (2009) [144]	Addiction (5; all males, range from 35 to 55 years)	NAc	EtOH	130	90	3.5–4.5 V	Bilateral Initial transient hypomania	None reported	All patients reported complete absence of craving for alcohol Follow-up was up to 8 years (2 died after 4 and 8 years) Two remained abstinent for years and 3 showed a reduction of consumption
Ge et al. (2018) [107]—5 patients overlap with Chen et al. (2019) [12]	Addiction (7; 6 × M and 1 × F, range from 26 to 50 years)	NAc + ALIC	Heroin	145 (NAc) 185 (ALIC)	150–240	2.0–3.3 V	Bilateral	None reported	Abstinence ranges from 3 months (lost to follow-up) to at least 40 months (4 out of 7 have been abstinent for > 20 months); mood also improved for most patients Pre-DBS LFP: Both NAc and ALIC showed prominent theta and alpha band activity A distinct beta band peak was also detected at ALIC There was a significant negative correlation between the power of theta band of ALIC and craving score, and a positive correlation between the power of alpha band of NAc and scores on the Hamilton depression inventory LFPs of the NAc and ALIC exhibited higher coherence in the theta and alpha bands
Zhang et al. (2018) [132]	Addiction (1; 39 M)	VC/VS	Heroin	130	90	3.5 V	Bilateral	Hypomania Death secondary to overdose (after relapse)	Comorbid antisocial personality disorder (baseline) Initial improvement but multiple relapses despite altering DBS parameters
Chen et al. (2019) [12]—5 patients overlap with Ge et al. (2018) [107]	Addiction (8; 7 × M and 1 × F, range from 22 to 50 years)	NAc + ALIC	Heroin	130–185	150–240	1.5–7 V	Bilateral	One intracranial hemorrhage (< 3 mL) with no neurological deficit Transient fever, headache, insomnia, and subjective slight memory decline	Some had comorbid depression and OCD Five patients were abstinent for more than 3 years, and the other 2 relapsed after abstaining for 6 months 1 was lost of follow-up at 3 months Craving reduced if the patients remained abstinent. Simultaneous DBS of NAc and ALIC also improved the quality of life, alleviated psychiatric symptoms, and increased glucose metabolism in certain addiction-related brain region (e.g., inferior frontal gyrus) on PET scan
Ge et al. (2019) [147]	Addiction (2; 38 M, 49 M)	NAc	Methamphetamine	150, 165	210, 240	2.5, 3.3 V	Bilateral	Insomnia, hypomania, teeth grinding at higher voltages	Only one out of two patients remained abstinent (at 1.5 to 2.5 years), and reviewing post-operative CT and pre-operative MRI showed incorrect placement of lead in the subject who relapsed
Leong et al. (2020) [75] *Cortical implant*	Addiction (8; 4 × M and 4 × F, 20–65 years)	ACC (rostrodorsal)	EtOH (refractory to multiple treatments)	6 or 10	NA	NA	Bilateral Mode: Burst (7) or tonic (1)	Infection (2), seizure (1), venous infarct (1), psychosis (1), impulsive behavior (1)	The self-reported alcohol craving reduced by 61% (end point ranged from 12 to 48 weeks, depending on presence of adverse events) Post-stimulation led to reduction in in current density at the rdACC for beta-1 band (13–18 Hz) on EEG
Sildatke et al. (2020) [148]	Addiction (4; all males, range from 24 to 57 years)	NAc	Opioid	NA	NA	NA	Bilateral	It appeared DBS was not turned on but the electrodes were used for LFP recording	EEG and LFP studies showed significant accuracy-related modulations in the LFPs at the time of the error-/correct-related negativity in 2 patients and at the time of error-positivity in 3 patients (Eriksen flanker task)
Zhang et al. (2020) [91]	Addiction (1; 42 M)	NAc	Heroin	NA	NA	NA	Bilateral	None mentioned	Reduction in drug cravings and remained drug-free (except for 1 episode at 6 months) at 1 year Implants were removed at the end of treatment (at patient’s request)
Zhu et al. (2020) [68]	Addiction (1; 28 M)	NAc DBS combined with anterior capsulotomy	Bucinnazine, morphine, hypnotics (13 years)	160	90	2.70, 2.75 V	Bilateral	None reported	Comorbid depression and anxiety disorder Reduced craving since 3 months and abstinent for at least 1 year Improvement in sleep, cognition, depression/anxiety, QoL seen
Mahoney et al. (2021) [149]	Addiction (1; 30sM)	NAc/ALIC	Opioid and BZ	145	90	6 V	Bilateral	None	Abstinent at 12 months Improved subjective craving, mood, frontal, and executive function PET scan showed an increase in glucose metabolism in the dorsolateral prefrontal and medial premotor cortices

The contradicting evidence above may be explained by the large activation volume of stimulation pulses, such that pulses centered on the core may also affect the shell, and vice versa. From human studies, the volume of activation was estimated to be between 30 and 120 mm^3^ [60] and the human NAc volume is around 700 mm^3^ [61]. However, for adult Sprague–Dawley rats, the total volume of NAc is approximately 5 mm^3^ with the NAc core making up half of that [62]. Therefore, neighboring structures could be co-stimulated in rats. Notably, the core and shell divide is functionally present in humans while they are less distinct in rodents [63, 64].

The second issue concerns laterality. While most studies used bilateral stimulation, some studies only used unilateral NAc stimulation [34, 39, 41, 43]. One study used left, right, and bilateral NAc core stimulation, demonstrating only the latter two were effective in reducing heroin seeking behavior [41]. It is unclear why such asymmetry exists. Dopamine concentration in the NAc is reported to be higher on the side ipsilateral to the paw-dominance of a mouse [65]; however, morphometric NAc human studies based on laterality have been controversial [49].

Almost all of these published studies in humans show some improvement from DBS in the NAc in addiction-related behaviors (Table 2) as well as cognitive improvement, which raises the possibility of potential publication bias. With this precaution in mind, NAc DBS is likely the lowest hanging fruit for refractory addictive disorders. A recent systematic review supports NAc DBS to treat substance use disorders, demonstrating that NAc DBS in patients led to a relapse rate of ~ 39% with a reasonable safety profile, which is an improvement compared to the ~ 60% relapse rate reported in that population (selection bias factors notwithstanding) [66]. In addition, individual case reports revealed that those with comorbid psychiatric disorders, such as depression and OCD, showed improvement in these other symptoms with NAc DBS with or without anterior limb of internal capsule (ALIC) intervention (DBS or capsulotomy) [12, 67, 68]. DBS may provide additional benefit for patients with multiple psychiatric comorbidities.

### Cortical Stimulation

The PFC is believed to provide top-down control of NAc in addiction (Fig. 2). DBS of the PFC (subregion not specified) significantly reduced cocaine seeking and motivation to take cocaine measured by progressive ratio test [35]. Stimulation of the infralimbic cortex of the PFC following a cocaine prime also reduced cocaine reinstatement [47]. Notably, mPFC stimulation in rats showed increased phasic dopamine release in the NAc, which might explain the stimulation effect [69]. However, stimulation of the infralimbic cortex starting 1 h or 4 days before alcohol prime did not affect alcohol-induced relapse following abstinence in rats [44]. The contrasting findings may be due to the different mechanisms of actions of the drugs being given (alcohol is a depressant whereas cocaine is a stimulant). Alternatively, infralimbic cortex stimulation may be more effective in reducing relapse-like behaviors following extinction rather than forced abstinence, consistent with the well-known role of the infralimbic cortex in extinction [70]. Infralimbic cortical DBS success is concordant with reduced craving by transcranial direct current stimulation (tDCS) of dorsolateral PFC in humans [71]. However, DBS of the prelimbic cortex following cocaine priming had no effect on cocaine reinstatement in rats [47].

In the OFC, DBS prevented rats from learning morphine-induced CPP, while DBS during extinction facilitated extinction acquisition and prevented morphine-induced reinstatement [9]. Chronic DBS of the anterior insular cortex either during 11 days of extinction or 14 days of abstinence reduced CPP, but CPP relapsed 10 days after chronic DBS [57]. This finding is consistent with how repetitive transcranial magnetic stimulation (rTMS) reduced heavy drinking only during the intervention but not after in alcohol-dependents [72]. The insula’s superficial location and delicate anatomy may be better suited for non-invasive interventions such as rTMS rather than DBS.

Anterior cingulate cortex (ACC) dysfunction can disrupt ongoing processing and detection of erroneous outcomes relevant for reward learning [73, 74]. ACC DBS following cocaine prime did not alter reinstatement following self-administration in rats [47]. However, when people with alcohol dependence received bilateral DBS (6- or 10-Hz stimulation), craving was significantly reduced [75]. The reduced craving was associated with a reduction in beta-1 band (13–18 Hz) current density on electroencephalogram (EEG) post-stimulation in the rostrodorsal ACC [75]. Alcohol craving also correlated with beta activity in the dorsal ACC in a case report, although relapse was associated with increased gamma band activity [76]. Another case report showed that a patient with concomitant OCD and alcohol dependence was successfully treated with ACC rTMS [77]. Interestingly, NAc DBS reduced craving correlated with improvement in error-related negativity in LFP in the ACC in humans [78], suggesting the effect of NAc DBS may alleviate ACC dysfunction. In addition to DBS, rTMS of the dorsal ACC demonstrated improvement in alcohol craving and concomitantly reduced NAc activation on fMRI [76]. Taken together, DBS in ACC may be particularly relevant for alcohol use.

Most of these cortical regions project to the NAc, raising the possibility that their DBS effects are mediated via these connections [49, 79]. Animal studies show not only strong projections from the cortex to NAc, but also a high degree of interplay between the two regions [80]. For example, *c-Fos* reactivity is observed in infralimbic cortex and NAc during DBS [42, 56] Pharmacological silencing of cortical interneurons can reinstate drug seeking [42, 81], which is consistent with silencing effects observed in the NAc [81]. Nevertheless, NAc is unlikely to be the sole driver of effects of DBS in the PFC considering that DBS in the prelimbic cortex does not affect drug seeking in rats, even though prelimbic cortex has strong connections to NAc [47].

### STN Stimulation

Parkinson’s patients who take dopaminergic medications for their movement disorder may chronically feel compulsion to over-consume such agents, even at the expense of dyskinesia and other side effects. Studies suggest that STN DBS in humans may improve movement symptoms as well as reduce compulsive use of medications [82, 83].

STN DBS or lesions in rats have been shown to reduce motivation for cocaine taking, while increasing motivation to consume more common rewards such as food [10, 37, 59, 84]. The lesion study in particular suggests that STN DBS may work via local inactivation [59, 84]. Further evidence shows STN HFS in rats inhibiting substantia nigra, entopeduncular nucleus, and NAc shell measured with brain mapping analyses of immediate-early gene expression but not PFC, and silencing STN neurons measured using recording ex vivo [37].

Although HFS of STN in rats reduced both cocaine taking and heroin seeking [37, 59], a conflicting study demonstrated only LFS (30 Hz) but not HFS modulates cocaine taking behavior in the presence of foot-shock punishment [10]. Abnormal alpha/theta and low-beta oscillatory activity during escalation of the cocaine intake phase also predicted the subsequent emergence of compulsive-like seeking behavior, where the animals were shock-resistant [10]. The discrepancy here may be explained by the absence/presence of punishment during drug taking, but further studies are warranted to understand STN LFS vs HFS outcomes.

### Other Brain Regions

Electrical stimulation targeting other brain regions in animals—amygdala [47, 85], hippocampus [47], periaqueductal gray matter [54], lateral hypothalamus [35], dorsal striatum [40, 44], and lateral habenula [58]—has also been described in the literature (Table 1). However, due to the limited number of studies per brain region, meaningful conclusions are difficult to draw. Hence, these regions are beyond the scope of this review.

### High- Versus Low-Frequency Stimulations

Whereas most animal studies reported HFS (> 130 Hz) effects on behavior, some used a lower frequency (< 20 Hz) and observed beneficial effects targeting the NAc [43], lateral habernula [58], ventral striatum [56], and periaqueductal gray [54] even at frequencies as low as 8 Hz (STN) [10] (Table 1). In contrast, LFS targeting the NAc shell [33], PFC [35], or OFC [9] were ineffective. Interestingly, DBS at 12 Hz alone did not affect cocaine-induced locomotor sensitization, but combined with dopamine D_1_-R antagonist, reversed cocaine-induced hyperlocomotion and plasticity in the NAc shell [33]. Such findings suggest that LFS may be effective only when basal dopamine signaling is low.

The effect of HFS in NAc shell may be short-lived, lasting less than 4 h in a cocaine experimenter-administered mouse model [33] or 1 day in an alcohol self-administration rat model [34]. However, reduction in cue-induced seeking behavior was maintained more than 4 weeks post-HFS (NAc shell) compared to the sham group in rats that self-administered methamphetamine [45]. Yet, LFS and HFS (mixture of NAc core and shell) effects lasted at least 2 weeks but less than 30 days in a cocaine self-administration rat model [43]. The different DBS parameters, targets, drugs, rodents, and tests across these studies (Table 1) pose a challenge to evaluate the best DBS protocol to reduce drug use.

### Potential Mechanism(s) of Action

#### Neuronal Inhibition

Despite the promising results of DBS in addiction, the mechanism of action is poorly understood and likely highly complicated. Several hypotheses on the mechanism of DBS in addiction have been suggested, such as direct inhibition/excitation of neural activity, information lesioning, and synaptic filtering where DBS blocks transmission of pathological low-frequency oscillations [86]. In particular, the inhibition hypothesis is supported by animal studies where both injection of GABA agonists (i.e., temporary inhibition) and HFS in the NAc was found to reduce alcohol use in rats [34]. Similar effects were also seen between STN lesion paradigms and DBS with cocaine conditioning [59, 84]. It is likely the neurons and their respective synaptic activities respond in a combination of different ways depending on the stimulation frequency and the drug being tested.

#### Depotentiation of Excitatory Inputs onto D_1_-R Via Modulation of Glutamate Receptors

Evidence also suggests the interaction between dopamine and glutamate receptors is involved in the mechanism of DBS action. For example, NAc shell LFS (12 Hz), only when used in combination with a D_1_-R antagonist, could reverse the motor sensitization and cocaine-evoked plasticity changes in D_1_-Rs on GABAergic MSNs, in the form of increases AMPA/NMDA glutamate receptor ratio [33]. By using a metabotropic glutamate receptor (mGluR1) antagonist to block the therapeutic effect, the authors proposed that the mechanism of action of DBS is mGluR-dependent, the activation of which depotentiates excitatory synaptic inputs onto D_1_-Rs on GABAergic MSNs [33]. Such depotentiation was associated with a normalization in drug-adaptive behavior. The findings are also consistent with the upregulation of GluR1 seen in the NAc in other stimulation studies [35, 36].

In contrast, both LFS (20 Hz) and HFS (160 Hz) stimulation of the NAc core attenuated cocaine seeking behavior in rats without the need of additional medications [43], similar to the outcome of PFC stimulation [35]. The contrasting findings between NAc core and PFC stimulations may be explained by the different levels of basal dopamine in these brain regions. Examining how DBS affects mGluR1 expression in different regions to affect D_1_-R signaling would be informative in future work.

#### Antidromic Activation of Cortex

Post-mortem brain *c-Fos* immunohistochemistry following in vivo NAc shell HFS showed both local activation *and* activation of the infralimbic cortex, suggesting that DBS of the NAc shell led to antidromic stimulation of the cortex via cortico-accumbal afferents [42]. Consistent with infralimbic cortex-NAc circuitry in DBS, electrophysiological studies showed NAc HFS but not LFS enhances both NAc-evoked LFP responses and spontaneous LFP slow oscillatory activity in OFC in an antidromic fashion [87]. These results further demonstrate the complex interplay between NAc and the cortex.

#### Dopamine Replacement

One study showed that HFS of the NAc shell led to a paradoxical augmentation in relapse behavior in a rat model of alcohol addiction [44], a contrary finding to other studies (Table 1). The authors also demonstrated an increase in extracellular levels of dopamine as measured by microdialysis. DBS-evoked increase in dopamine is reminiscent of the intracranial self-stimulation model, where the subjects self-administer electrical stimulation via implanted electrodes in the brain [88]. Reinforcing effects of electrical stimulation is presumably mediated by dopamine release in the reward circuitry [89]. In addition, medial PFC DBS evoked dopamine release in the NAc in a frequency-dependent manner in anesthetized rats measured by fast-scan cyclic voltammetry (FSCV) [69]. Taken together, DBS of PFC may operate partially via the replacement of dopamine, but whether this can reduce drug taking or seeking has yet been tested. Most of the DBS studies did not employ in vivo microdialysis techniques for direct quantification; hence, replacement of dopamine as a DBS mechanism is unconfirmed.

### Limitations of Animal Models

Human subjects tend to require weeks and months of DBS to observe an improvement, whereas animal studies show an almost immediate effect. In animal studies, the conditioning occurs over days (rather than years). The short timeframe indicates that the neuroplasticity reversal may not be the dominating effect of DBS in animals and the dopamine replacement caused by stimulation may mediate the acute improvement in behavior. In humans, DBS likely operates via reversal of neuroadaptations that have been built up on a much longer time scale than animals subjected to conditioning. This interpretation is supported by case reports where subsequent explantation of the DBS device did not reverse the initial effect of abstinence [90, 91]. In addition, a further case report showed that NAc DBS reduced risk taking behavior and increased activation of brain regions implicated in behavioral control arising from lasting neuroadaptations, measured by positron-emission tomography [92].

Some human studies also include subjects with comorbid OCD, depression, and anxiety [12, 93, 94]. In fact, addiction may be part of a maladaptive coping mechanism for other underlying disease/s. DBS may alleviate addiction by reducing the severity of an underlying comorbid disease, which is challenging to model in animals. For example, the higher cognitive and emotional component as well as the impact of social cues are difficult to assess in animals.

### Alternatives to DBS

Given the invasive nature of DBS, other neuromodulatory modalities are worth considering. Of note, noninvasive techniques such as rTMS and tDCS have been of particular interest [95, 96]. A detailed review of these techniques and outcomes is out of the scope of this article but in brief, rTMS operates by applying a stimulating coil at the scalp, which generates magnetic fields. The magnetic fields then pass through the skull and induce strong focal currents in the underlying brain tissues [97]. It is believed that long-term repetitive stimulation can lead to neuroplasticity, which can provide therapeutic effect to the addicted brain [97]. tDCS involves application of a direct or alternative electrical current to the scalp, such that current travels from a positive (cathode) to a negative (anode) electrode. Cortical neurons are suggested to be facilitated under the anode, whereas those under the cathode are suppressed [98]. Although rTMS and tDCS have been shown to be effective in preclinical studies and small clinical case series on reducing symptoms such as craving, more data are required to ascertain their long-term safety profiles, efficacy, and the ideal patient profile [98–100]. Although research shows that non-invasive neuromodulation can be successful in certain cases without the need for neurosurgery, the main limitations are the focality of these non-invasive treatments and the relatively low spatial resolution. These non-invasive interventions also require numerous frequent visits to facilities in a specialized, tertiary center. In contrast, DBS can be implanted as a permanent or chronic stimulation system, which can potentially be programmed remotely [101].

There was a recent success of semi-invasive MRI-guided focused ultrasound (MRgFUS) thalamotomy in the treatment of movement disorders and other psychiatric disorders (depression and OCD) [102, 103]. Such a technique would avoid the complications associated with the implantation of devices, which may necessitate explantation for reasons such as lack of efficacy or infection. Alongside DBS, MRgFUS offers a novel therapeutic avenue that should be explored to treat refractory addictive disorders.

Invasive techniques can offer a more focal region of therapy. Ablation techniques in drug addiction targeting the NAc with promising results have long received attention [104]. Indeed, a recent systematic review showed that NAc DBS has similar efficacy and safety profile as radiofrequency ablation of the NAc [66]. Compared to ablation techniques, however, DBS allows adjustment of stimulation parameters tailored to maximize therapeutic efficacy while minimizing side effects. Nonetheless, a recent systematic review showed that NAc DBS has similar efficacy and safety profile as radiofrequency ablation of the NAc [66].

### Future Directions

#### Potential Biomarkers

The hunt for a reliable biomarker serves three critical purposes: to facilitate patient selection, to monitor the progress of the disease (and therapeutic effect of treatments), and to progress towards a holy grail of DBS—a closed-loop therapeutic system. Not only can this potentially increase the battery longevity (hence reducing the need for battery replacement), it also can increase the efficacy of stimulation by tailoring to the patients’ neural responses. Further, a “tolerance” effect can be observed with chronic open-loop DBS stimulation in movement disorder patients, with the stimulation effect wearing off after a period of time [105]. Such a tolerance is also likely in patients receiving DBS to treat psychiatric illnesses. Closed-loop system with intermittent, dynamic stimulation can potentially minimize tolerance. Such biomarkers need to be easy to measure and possess a relatively high spatiotemporal resolution of measurement that, in turn, correlates well with clinical/behavioral changes. In our opinion, the most promising candidates would be electrophysiological and neurochemical measurements, although they will need to be tailored towards individual addictive substances and patients.

In terms of electrophysiological measurements, LFP is the most widely studied potential biomarker among DBS studies. LFP’s wide use is mostly because of its measurement using the stimulating electrodes themselves. In urethane-anesthetized rats, NAc HFS suppressed pyramidal cell firing and enhanced slow LFP oscillations in the OFC via antidromic activation of cortico-striatal recurrent inhibition [87]. In addition, prolonged NAc HFS and LFS (delivered over 90 min) have been shown to affect both spontaneous and evoked LFPs in the medial PFC, lateral OFC, mediodorsal thalamus (MD), and NAc sites [106]. HFS also produced widespread increases in spontaneous beta and gamma power and enhanced coherent beta activity between MD and all other regions. In contrast, LFS elevated theta power in the MD and NAc. Acutely, HFS increased and LFS decreased induced relative gamma coherence. Stimulation-induced changes in gamma coherence shows that NAc DBS may achieve therapeutic effects by restoring the synchronicity of a neural circuitry that is disrupted in neuropsychiatric diseases. The differences in the effect on LFPs between HFS and LFS may also explain the differences in behavioral effects described above.

In a study assessing 7 people with heroin use disorder, pre-DBS electrophysiology revealed prominent theta and alpha band activity in both the NAc and ALIC [107]. Additionally, a beta band peak was detected in the power spectra of ALIC LFPs, which, the authors hypothesized, may represent the activity of striatal bridge cells. Furthermore, there was a negative correlation between the power of the theta band of ALIC LFPs and subjective craving, and a positive correlation between the power of the alpha band of NAc LFPs and depressive symptoms. LFPs of the NAc and ALIC both exhibited higher coherence values in the theta and alpha frequency bands. No comparison with post-DBS recordings was given in this study. Together with the animal and human DBS studies on NAc [108, 109], the most consistent biomarker frequency is yet unknown. Further studies are required for clarification. New technologies such as the Medtronic Percept Stimulator [110] may be able to provide more information by simultaneously recording from and stimulating brain targets in humans. In addition, trials investigating electrophysiological changes secondary to DBS in addiction (such as [111]) will provide more insights.

Unlike electrophysiological signals (i.e., action potentials) that naturally lead to neurotransmitter release into synapses, quantification of synaptic-related neurotransmitter extracellular concentrations directly measures the activation of relevant neural circuits (such as the ones depicted in Fig. 2). Most studies in addiction focus on the monoamines, particularly dopamine, glutamate, and 5-HT. One animal study reviewed here [44] employed in vivo microdialysis and found that local dopamine concentrations increased after NAc shell HFS. While in vivo microdialysis has long been the gold standard for sampling neurochemicals, the process necessitates an external analyzer and has low temporal resolution. In contrast, in vivo electrochemical techniques, such as FSCV, provide potential benefits such as higher temporal resolution, less tissue damage, and the ability to be chronically implanted [112–116]. However, compared to microdialysis, electrochemical techniques have other limitations. Often, they are restricted to measurement of a single electroactive amine at a time, such as dopamine or serotonin. Thus, future studies which aim to develop biomarkers for DBS efficacy will need to demonstrate suitability of neurochemical biomarkers for translation and may be limited by present detection technology. While microdialysis provides important measurements of the tonic (basal) extracellular levels of neurochemicals, voltammetry can provide measurement of phasic and tonic measures of neurochemicals [117–122].

#### Future Preclinical and Clinical Research Considerations

Other important considerations for future research include inclusion of both genders in both animal and human studies (currently most studies focus on males—see Tables 1 and 2), and the use of larger animals—preclinical studies are predominantly rodent-based, but also well suited to large animal models. In addition, we have focused on technologies that are currently clinically viable in this review, namely electrical DBS devices. However, the use of optogenetics, where a viral vector is able to transfect a specific neural population, is increasingly popular in the preclinical study of underlying pathophysiology of different neuropsychiatric disorders and relevant therapies. Optogenetics has provided new insights into DBS protocol optimization for Parkinson’s disease with translational potential [123, 124], which may be extended to psychiatric illnesses. In addition, recent optical imaging techniques allow combination of fiber photometry and electrophysiology to study specific cell population activity via genetically encoded calcium indicators, including during deep brain stimulation [125, 126]. Cell-specific viral transfection in calcium imaging or optogenetics helps to study specific ion channels or neurotransmitters. However, the use of viruses in humans is very limited. Further technological advancements are necessary to harness optical methods in the clinical setting.

Currently, the use of DBS in addiction remains off-label, primarily due to the lack of high-quality clinical evidence. As seen in Table 2, most human studies consist of observational studies with a small sample size. However, more clinical trials are currently being conducted. The *ClinicalTrials.gov* database show that there are currently around 10 DBS trials underway trying to tackle this paucity of scientific evidence, targeting the NAc, ALIC, and STN [127]. As demonstrated in the present review, they are all very promising targets. In addition, we would add PFC to this list given the success from animal studies [47] as well as human imaging studies showing its dysfunction in subjects with substance use disorder [128].

Supporting the potential of DBS in alleviating addiction-related behaviors, DBS to treat obesity has also shown early successes [129–131]. Given the similarities between substance use disorders and obesity, the findings in DBS for substance use disorder presented so far may be translatable to DBS for obesity. With the increasing prevalence and comorbidities associated with excessive calorie consumption in the modern society, this can be hugely beneficial.

While it is exciting to perform cutting-edge patient surgeries, it is also important to recognize that DBS is not an immediate cure and requires careful patient selection, consent, and patient-based optimization (including treating other comorbidities); otherwise, DBS can lead to a risk of major adverse outcomes [132]. DBS also has cost, translational, and capacity limitations for a highly prevalent disorder, but has the advantage of elucidating fundamental neural biology capable of detecting new therapeutic targets.

In our literature search, all human studies that involve stimulation of deep structures were conducted exclusively with high-frequency stimulation, as commonly used in DBS for movement disorders (see Table 2). Therefore, future work to compare how stimulation at different frequencies may affect the biomarkers such as LFP in humans would be beneficial.

The duration of trials may also be important. One of the possible reasons attributed to the failure of early trials in DBS for depression is the short treatment duration, as trials with longer duration appeared to show significant efficacy [6, 133–135]. DBS duration effects will need to be assessed in larger clinical trials. Further, for neuropsychiatric conditions such as addiction, careful consideration of the ethical aspect is required.

#### Ethical Considerations

Although DBS is much safer than early forms of psychosurgery such as lobotomy, it is not a risk-free treatment [136]. Therefore, clinicians are obliged to always have the patients’ best interest in their mind. The risks and benefits of the treatment need to be balanced appropriately. One of the main issues with human trials is the principle of autonomy, as patients with addictive disorders may not have the capacity to consent to such process. Furthermore, there are concerns that DBS (and indeed any forms of psychosurgery) may alter a patient’s personality, which can lead to actions and consequences that may otherwise not be performed [137–139]. Academic societies and government institutions should also provide a medico-legal framework to ensure both medical professionals and patients are protected. A full ethical discussion is out of the scope of this review. Nevertheless, we recommend that the clinical team be multidisciplinary, with access to the resources and expertise to assess and manage the patients before and after the DBS surgery.

## Conclusions

Preclinical and clinical studies suggest DBS is a potential treatment for refractory addictive disorders after other non-invasive options fail. Here, we reviewed these studies and their implications. However, given the uncertainty in its mechanism of action and a lack of large scale randomized controlled trials, clinicians and scientists should be cautiously optimistic. We also provided recommendations on the directions of future research, such as the need to identify reliable biomarkers, for which LFPs in the NAc and in vivo electrochemical measurement of monoamines so far appear the most promising options. With further research, we believe it can prove to be a highly effective treatment for a selected group of patients.

## Supplementary Information

Below is the link to the electronic supplementary material.

Supplementary file1 (PDF 516 KB)

Supplementary file2 (PDF 525 KB)

Supplementary file3 (PDF 542 KB)

Supplementary file4 (PDF 524 KB)

Supplementary file5 (PDF 516 KB)

Supplementary file6 (PDF 542 KB)

Supplementary file7 (PDF 560 KB)

Supplementary file8 (PDF 542 KB)

Supplementary file9 (PDF 533 KB)

Supplementary file10 (PDF 560 KB)

Supplementary file11 (DOCX 19 KB)

Supplementary file12 (PDF 607 KB)
