# Supplementary material for: Deep Brain Stimulation for Addictive Disorders—Where Are We Now?
Source: Neurotherapeutics. 2022 Apr 11;19(4):1193–215. doi: 10.1007/s13311-022-01229-4 (PMC9587163; doi:10.1007/s13311-022-01229-4)
Supplement: Supplementary file 11 — Supplementary file11 (DOCX 19 KB) [file 13311_2022_1229_MOESM11_ESM.docx]

Appendix A. Search criteria for literature review

A systematic database search was performed using Pubmed (Medline) and EMBASE, using the key words in the title and abstract. The search was performed on 06/18/2021.

The full search is listed as follows:

Database(s): **Ovid MEDLINE(R) 1946 to Present and Epub Ahead of Print, In-Process & Other Non-Indexed Citations and Ovid MEDLINE(R) Daily, Embase**1974 to 2021 June 18
Search Strategy:

| **#** | **Searches** |
| --- | --- |
| 1 | Deep Brain Stimulation/ or "Transcranial Magnetic Stimulation"/ or "Transcranial Direct Current Stimulation"/ or exp "noninvasive brain stimulation"/ or exp "brain depth stimulation"/ or exp "transcranial magnetic stimulation"/ or "transcranial electrical stimulation"/ |
| 2 | (((stimulation or "electro-stimulat*" or electrostim* or "electro-therap*" or electrotherap*) adj2 ("Nucleus Accumbens" or "Subthalamic Nucleus" or brain or cortex or cortical or transcranial or cranial)) or tDCS or (deep adj (rTMS or TMS))).ti. |
| 3 | 1 or 2 |
| 4 | exp *Substance-Related Disorders/ |
| 5 | *Alcoholism/ |
| 6 | *Alcohol-Related Disorders/ |
| 7 | Heroin Dependence/ |
| 8 | *Amphetamine-Related Disorders/ |
| 9 | *narcotic dependence/ |
| 10 | (addict* or alcoholic* or ((substance or drug or alcohol or opioid* or opiat* or opium or narcot* or heroin* or cocaine or amphetamin* or nicotine or cannabi* or hallucinogen*) adj (usage or abuse or consumption or addiction or disorder* or misuse*))).ti. |
| 11 | or/4-10 |
| 12 | 3 and 11 |
| 13 | (conference abstract or conference review or editorial or erratum or note or addresses or autobiography or bibliography or biography or blogs or comment or dictionary or directory or interactive tutorial or interview or lectures or legal cases or legislation or news or newspaper article or patient education handout or periodical index or portraits or published erratum or video-audio media or webcasts).mp. or conference abstract.st. |
| 14 | 12 not 13 |
| 15 | limit 14 to english language |
| 16 | remove duplicates from 15 |

Search using the same key words in the Cochrane library yielded 0 relevant results.

We excluded:

- non-English articles;
- review papers;
- editorials / opinion pieces;
- studies that use other stimulation techniques;
- studies that focus on diseases other than addictive disorders;
- studies relating to gaming or internet addiction;
- conference abstracts;
- human studies.

Titles and abstracts of all search results were reviewed. Included articles were further reviewed in full text.
